# Digital Quantum Simulation of Nonadiabatic Geometric Gates via Shortcuts to Adiabaticity

**DOI:** 10.3390/e22101175

**Published:** 2020-10-19

**Authors:** Yapeng Wang, Yongcheng Ding, Jianan Wang, Xi Chen

**Affiliations:** 1 International Center of Quantum Artificial Intelligence for Science and Technology (QuArtist) and Department of Physics, Shanghai University, Shanghai 200444, China; shuwgs@shu.edu.cn (Y.W.); jiananwang@shu.edu.cn (J.W.); 2 Department of Physical Chemistry, University of the Basque Country UPV/EHU, Apartado 644, 48080 Bilbao, Spain

**Keywords:** shortcuts to adiabaticity, nonadiabatic geometric phase, quantum gate, digital simulation

## Abstract

Geometric phases are used to construct quantum gates since it naturally resists local noises, acting as the modularized units of geometric quantum computing. Meanwhile, fast nonadiabatic geometric gates are required for reducing the information loss induced by decoherence. Here, we propose a digital simulation of nonadiabatic geometric quantum gates in terms of shortcuts to adiabaticity (STA). More specifically, we combine the invariant-based inverse engineering with optimal control theory for designing the fast and robust Abelian geometric gates against systematic error, in the context of two-level qubit systems. We exemplify X and T gates, in which the fidelities and robustness are evaluated by simulations in ideal quantum circuits. Our results can also be extended to constructing two-qubit gates, for example, a controlled-PHASE gate, which shares the equivalent effective Hamiltonian with rotation around the Z-axis of a single qubit. These STA-inspired nonadiabatic geometric gates can realize quantum error correction physically, leading to fault-tolerant quantum computing in the Noisy Intermediate-Scale Quantum (NISQ) era.

## 1. Introduction

A quantum computer based on a quantum gate and quantum circuits is one of the most promising solutions to the arising demand for computational resources, which is so-called digital quantum computing [1]. Quantum gate, as the basic operation, is modularized to evolve unknown quantum states by a time-dependent Hamiltonian, the design of which is highly related to quantum control. Specifically, there are two main difficulties in experimental technologies: (i) the manipulation of the quantum system is realized by external fields, where their imperfection introduces systematic errors; (ii) the quantum system is coupled to the noisy environment, which causes the decoherence. Among the theoretical frameworks, geometric quantum computation suggests that the geometric phase [2,3,4,5,6], as a property of fundamental quantum theory, can be employed to construct quantum gates, being naturally robust against noises [7,8,9,10]. The geometric phase can be a real number known as the Berry phase [2], or a non-Abelian matrix [3] that induces holonomy in the quantum theory. Therefore, quantum error correction and fault-tolerant quantum computers [11,12,13] can also be achieved by this protocol, boosting the development from NISQ gate-based quantum computers to the next level. Geometric quantum computation has been originally proposed to accumulate a geometric phase adiabatically, requiring long operation time so that the quantum information can be destructed by decoherence. Thus, nonadiabatic geometric quantum computation aims at shortening the operation time without loss of fidelity, which is experimentally demonstrated [8,14,15,16,17,18,19] in both Abelian [20,21,22,23] and non-Abelian cases [24,25]. However, the systematic errors induced by inaccurate driving fields harm the performance of geometric gates [26,27], which should be further optimized by quantum control techniques. Recently, both theoretical [28,29,30,31] and experimental researches [32,33] have been devoted to improving the robustness of (non-)Abelian gates against systematic errors. A typical framework NHQC+ [29] constructs a single-looped nonadiabatic geometric gate in an extensible way, being compatible with most optimal control methods for a balance between flexibility and robustness.

In a slightly different but relevant topic, “shortcuts to Adiabaticity” (STA), has been developed in the past decade [34,35], sharing the merits of adiabatic passages and resonant pulses for accelerating state evolution, but keeping the high-fidelity. Among all the techniques of STA, counter-diabatic (CD) (or equivalently quantum transitionless algorithm) [36,37] and invariant-based inverse engineering [38] are most popular from the viewpoint of theory and experiment. Regarding CD driving, the supplementary interaction is required to suppress the diabatic transition, thus following the adiabatic evolution of the previous reference Hamiltonian [36,37]. In addition, inverse engineering can be employed for designing nonadiabatic evolution along one of the dynamical modes of Lewis–Riesenfeld dynamical invariant [39] with boundary conditions [38]. As an extension, the freedom left provides the flexibility, allowing the suppression of undesired errors by incorporating with the optimal control [40,41], dynamical decoupling [42], and machine learning [29,43,44]. Though both methods are mathematically equivalent, the physical implementations are totally different [45]. In the context of geometric phase, CD driving helps mimic the adiabatic Berry geometric phase within shorter time, but inverse engineering provides the nonadiabatic Aharonov–Anandan geometric phase, relevant to the Lewis–Riesenfeld phase.

We reckon that the Abelian geometric gate is more feasible for experimental implementation, which can be applied in the superconducting circuit, which indeed consists of two-level subsystems, as a state-of-the-art quantum computing platform for its balance between scaling-up and experimental control. Thus, we model a qubit by Jaynes–Cummings Hamiltonian, which can be simplified by rotation wave approximation that neglects high-order oscillations. With time-dependent perturbation theory and Lewis–Riesenfeld theory, we inversely design the protocols for fast and robust Abelian geometric gates. Different from the theoretical proposals in References [28,29,30,31], we further apply the quantum circuit as an ideal simulator for implementing the digital quantum gates and thus evaluating their performances. For completeness, we also compare the counter-diabatic (CD) driving for the same proposal. Moreover, we introduce the extension to two-qubit geometric gates for generating the universal gate set. We hope our results can be useful to speed up digitalized adiabatic quantum computing, emerged with the digital-analog concept.

The paper is organized as follows. In Section 2, we introduce the model, Hamiltonian and STA designed from the Lewis–Riesenfeld invariant theory, by repeating the necessary results in References [28,29,30,31] for consistency. Later in Section 3, we implement the quantum gates in the digital simulator of superconducting circuits, and illustrate the improved performance of nonadiabatic geometric gates designed from STA. We compare the results with the method of CD driving in Section 4, claiming that all results can be extended to two-qubits. Finally, the paper is briefly concluded in Section 5.

## 2. Model, Hamiltonian, and Method

Digital quantum computing with a superconducting circuit can be modeled by Jaynes–Cummings (JC) Hamiltonian,

(1) $$H_{\mathrm{JC}}=\hbar \omega a^{†}a+\frac{\hbar }{2}\omega_{0}\sigma_{z}+\hbar g\left(a+a^{†}\right)\left(\sigma^{+}+\sigma^{-}\right),$$

where *a* and $a^{†}$ are the annihilation and creation operators of a harmonic oscillator with frequency ω, $\sigma_{z}$ and $\sigma^{\pm }$ are Pauli operators corresponding to the two-level system with ground state |0〉, the excited state |1〉, as well as the transition frequency $\omega_{0}$. With rotation-wave approximation (RWA), the dynamics of the JC model become solvable, remaining feasible for experiments at the same time. Specifically, by assuming $|\omega -\omega_{0}|\ll \omega +\omega_{0}$, counter-rotating terms, $a^{†}\sigma^{+}+a\sigma^{-}$, can be ignored, which the solvable Hamiltonian reads

(2) $$H_{\mathrm{JC}}=\hbar \omega a^{†}a+\frac{\hbar }{2}\omega_{0}\sigma_{z}+\hbar g\left(a\sigma^{+}+a^{†}\sigma^{-}\right).$$

For the construction of a geometric single-qubit gate by time-dependent external field, we choose the two lowest levels, |0〉 and |1〉, as the computational bases, with the dynamics governed by

(3) $$H\left(t\right)=\frac{\hbar }{2}\left(\begin{array}{cc} 0 & \Omega \left(t\right)\exp\left[i\phi (t)\right]\\ \Omega \left(t\right)\exp\left[-i\phi (t)\right] & 0 \end{array}\right),$$

which is the simplified Hamiltonian in subspace {(|0〉and|1〉}. According to the Lewis–Riesenfeld theory [39], the dynamical invariant with units of energy,

(4) $$I\left(t\right)=\frac{\hbar }{2}\Omega_{0}\left(\begin{array}{cc} \cos\theta & \sin\theta \cos\beta -\sin\theta \sin\beta \\ \sin\theta \cos\beta +\sin\theta \sin\beta & -\cos\theta \end{array}\right),$$

should satisfy the condition:

(5) $$\frac{dI(t)}{dt}=\frac{\partial I(t)}{\partial t}+\frac{1}{i\hbar }\left[I(t),H(t)\right]=0,$$

giving the following coupled auxiliary equations:

(6) $$\begin{array}{ccc} \dot{\theta } & = & -\Omega (t)\sin(\beta +\phi ), \end{array}$$

(7) $$\begin{array}{ccc} \dot{\beta } & = & -\Omega (t)\cot\theta \cos(\beta +\phi ). \end{array}$$

The eigenstates of the invariant are $|\psi_{+}\left(t\right)\rangle ={\left(\cos\frac{\theta }{2}e^{-i\frac{\beta }{2}},\sin\frac{\theta }{2}e^{i\frac{\beta }{2}}\right)}^{T}$, and $|\psi_{-}\left(t\right)\rangle ={\left(\sin\frac{\theta }{2}e^{-i\frac{\beta }{2}},-\cos\frac{\theta }{2}e^{i\frac{\beta }{2}}\right)}^{T}$, which describe the wave function by $\Psi \left(t\right)=\sum_{\pm }c_{\pm }\exp\left(i\gamma_{\pm }\right)|\psi_{\pm }\rangle$, with $\gamma_{\pm }$ being the Lewis–Riesenfeld phases. For simplicity, one can choose the state evolution along one of the dynamical modes. We denote the Lewis–Riesenfeld phase of one of two dynamical modes, $|\psi_{+}\left(t\right)\rangle$, by $\gamma_{+}\left(t\right)=\gamma_{G}\left(t\right)+\gamma_{D}\left(t\right)$, which consists of geometric and dynamical phases, that is,

(8) $$\begin{array}{ccc} \gamma_{G}\left(t\right) & = & \frac{1}{\hbar }\int_{0}^{t}\langle \psi_{+}|i\frac{\partial }{\partial t^{\prime }}|\psi_{+}\rangle dt^{\prime }=\frac{1}{2}\int_{0}^{t}\dot{\beta }\cos\theta dt^{\prime }, \end{array}$$

(9) $$\begin{array}{ccc} \gamma_{D}\left(t\right) & = & -\frac{1}{\hbar }\int_{0}^{t}\langle \psi_{+}\left|H\left(t\right)\right|\psi_{+}\rangle dt=\frac{1}{2}\int_{0}^{t}\dot{\beta }\sin\theta \tan\theta dt^{\prime }. \end{array}$$

By rephrasing Equation (6) and dividing Equation (6) by Equation (7), we get the equations for inverse engineering of the driving Hamiltonian

(10) $$\begin{array}{ccc} \Omega (t) & = & -\frac{\dot{\theta }}{\sin(\beta +\phi )}, \end{array}$$

(11) $$\begin{array}{ccc} \phi (t) & = & \arctan\left(\frac{\dot{\theta }\cot\theta }{\dot{\beta }}\right)-\beta , \end{array}$$

where the angular parameters θ(t) and β(t) are designed for canceling the dynamical phase (9) at the end of a cyclic evolution of gate time *T*. Here, we clarify that the angular parameter β(t) is obtained from solving another auxiliary equation $\dot{\eta }\left(t\right)=-\dot{\beta }/\cos\theta$:

(12) $$\beta \left(t\right)=-\int \dot{\eta }\left[\theta (t),t\right]\cos\left[\theta (t)\right]dt,$$

where $\eta \left(t\right)=-2\gamma_{+}\left(t\right)$ is the global phase, being expanded by a series of θ(t). Accordingly, a single-loop evolution accumulates a geometric phase, resulting in the gate operator, $U\left(T\right)=\sum_{\pm }\exp\left(i\gamma_{\pm }\right)|\psi_{\pm }\left(0\right)\rangle \langle \psi_{\pm }\left(0\right)|$, yielding

(13) $$\begin{array}{c} U\left(T\right)=\left(\begin{array}{cc} \cos\gamma +i\cos\theta_{0}\sin\gamma & i\sin\gamma \sin\theta_{0}e^{-i\beta_{0}}\\ i\sin\gamma \sin\theta_{0}e^{i\beta_{0}} & \cos\gamma -i\cos\theta_{0}\sin\gamma \end{array}\right), \end{array}$$

where $\gamma =\gamma_{+}$ when the single dynamical model $|\psi_{+}\rangle$ is used here. As a consequence, U(T) finally gives a universal single-qubit gate.

## 3. STA Design and Digital Simulation

The universal gate performs a rotation of −2γ around the axis of $\left(\sin\theta_{0}\cos\beta_{0},\sin\theta_{0}\sin\beta_{0},\cos\theta_{0}\right)$ after a single-loop geometric evolution. Now we introduce the protocols for Z rotation and X rotation, as two cases for illustrative reasons. Firstly, we consider a rotation around the Z axis, in which the angular parameter should satisfy the boundary conditions: cosθ(0)=1 and cosθ(T)=1. We split the single-loop evolution into two parts, which cancel the dynamical phase at the end. We set the angle parameter θ(t) by

(14) $$\theta \left(t\right)=\pi \sin^{2}\left(\frac{\pi t}{T}\right),$$

which determines the approximated expansion of the global phase [40,46]:

(15) η(t)=2θ(t)−sin2θ(t).

This choice of global phase is capable of suppressing the systematic error in the Rabi frequency, see the discussion below. Boundary conditions on β(t) give a sudden jump of −γ at t=T/2, i.e., β(0)=0 and $\beta \left(T^{+}/2\right)=\beta \left(T^{-}/2\right)-\gamma$, leading to β(t) by integrating Equation (7), nullifying the dynamical phase as well. Eventually, the control pulses Ω(t) and ϕ(t) can be inversely designed by solving Equations (10) and (11). The rotation around the X axis is more complicated since the single-loop evolution consists of four parts. For accumulating the geometric phase and canceling the dynamical one, we have

(16) $$\begin{array}{c} \theta \left(t\right)=\left\{\begin{array}{cc} \frac{\pi }{2}\left[1+\sin^{2}\left(\frac{2\pi t}{T}\right)\right],\; & 0\leq t<\frac{T}{2},\\ \frac{\pi }{2}\left[1-\sin^{2}\left(\frac{2\pi t}{T}\right)\right],\; & \frac{T}{2}\leq t\leq T, \end{array}\right. \end{array}$$

and the boundary conditions of β(t) being

(17) $$\begin{array}{cc} \beta (0)=0, & \beta \left(T^{+}/4\right)=\beta \left(T^{-}/4\right)-\gamma ,\\ \beta \left(T^{+}/2\right)=\beta \left(T^{-}/2\right), & \beta \left(3T^{+}/4\right)=\beta \left(3T^{-}/4\right)+\gamma , \end{array}$$

where the expansion of the global phase (15) and derivation of β(t), Ω(t), and ϕ(t) remain the same.

**Figure 1:**
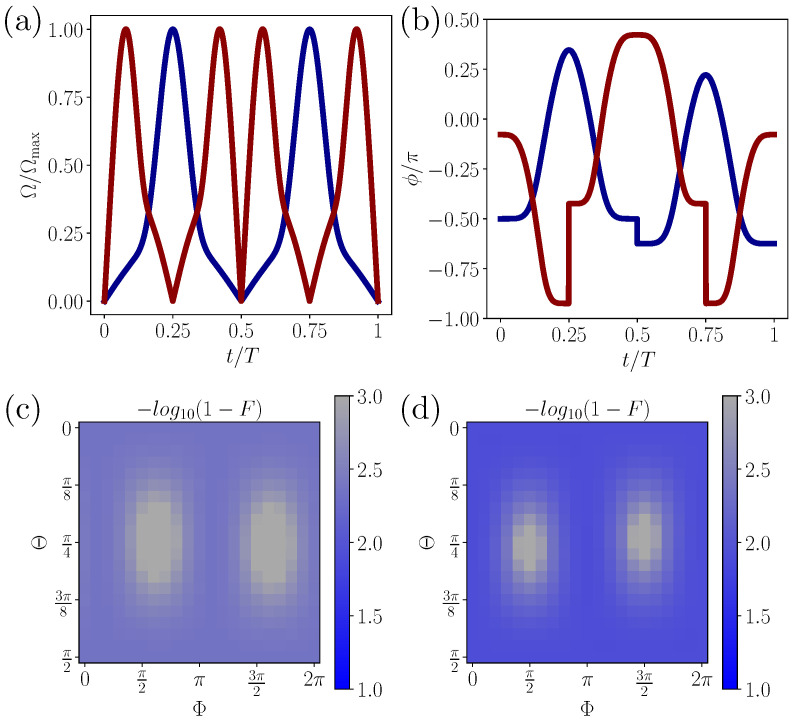
(**a**,**b**) Shapes of Ω(t) and ϕ(t) that characterize the time-dependent driving fields for constructing X gate (red line) and T gate (blue line). (**c**,**d**) Gate performance defined by $-\log_{10}\left(1-F\right)$, where *F* denotes the squared overlap between the ideal output and real output evolved by 50 Trotter steps, in which the fidelity is calculated for 21×21=441 inputs.

Now we implement two typical quantum gates as X gate and T gate by single-loop geometric evolution, which are realized by θ(0)=π/2,γ=π/2, and θ(0)=0,γ=−π/8, respectively, with β(0)=0 for both of them. For the experimental implementation, we bound the maximum Rabi frequency by $\Omega_{\max}=2\pi \times 20$ MHz, resulting in gate time *T* be 226 ns for X gate, and 324 ns for T gate. The pulses that construct nonadiabatic geometric gates are shown in Figure 1a,b. We evaluate the performance of them by choosing arbitrary inputs as ${\left(\cos\Theta ,\sin\Theta e^{-i\Phi }\right)}^{T}$, with figure-of-merit being fidelity, i.e., the overlap between the ideal target state and real final state. We simulate the dynamics of X and T gate operations in quantum circuits with 50 Trotter steps by ideal Rx and Ry gates, resulting in average fidelity ${\bar{F}}_{X}=0.991$ and ${\bar{F}}_{T}=0.997$ and minimal fidelity $F_{X}^{\min}=0.988,\;F_{T}^{\min}=0.996$, with the fidelities of arbitrary inputs shown in Figure 1c,d, where the Suzuki–Trotter expansion and other detailed techniques are given in Appendix A.

We emphasize that the protocols are robust against the systematic error of Ω-type, i.e., $\Omega \left(t\right)\to \left(1+\delta_{\Omega }\right)\Omega \left(t\right)$, since the expansion of the global phase is previously suggested. To be more specific, one can write down the first-order term of transition probability, following the time-dependent perturbation theory [40,46]:

(18) $$P=\frac{1}{4}{\left|\int_{0}^{T}\langle \Psi_{-}\left(t\right)|\left(\delta_{\Omega }\Omega \sigma_{x}\right)|\Psi_{+}\left(t\right)\rangle \right|}^{2},$$

where two orthogonal dynamical modes with LR phases included are denoted by $|\Psi_{\pm }\left(t\right)\rangle$. Substituting the auxiliary equations into Equation (18), we obtain the error cancellation condition

(19) $$\left|\int_{0}^{T}dte^{i\eta (t)}\dot{\theta }\sin^{2}\theta \right|=0.$$

**Figure 2:**
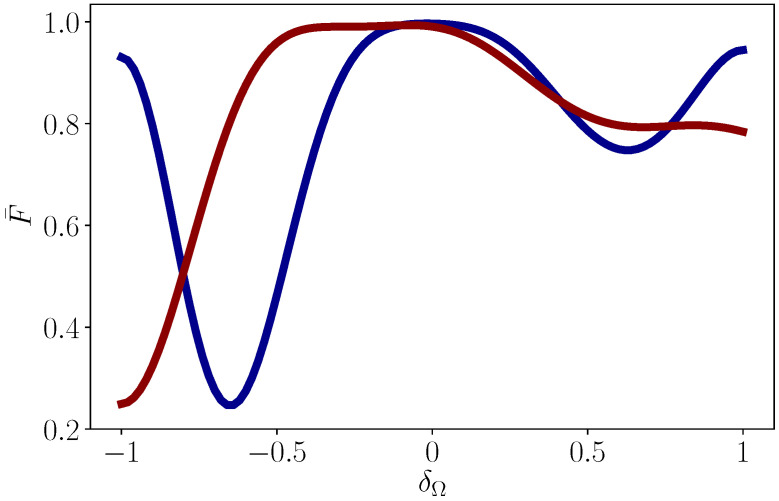
Average fidelity of X gate (red line) and T gate (blue line) versus Ω-error defined by $\Omega \left(t\right)\to \left(1+\delta_{\Omega }\right)\Omega \left(t\right)$. The average fidelity is approximated by averaging 441 values of fidelity instead of integrating Θ and Φ. Other parameters are the same as those in Figure 1.

Here we emphasize that the optimal solution in Equation (15) can be obtained numerically [46] and analytically from the Euler–Lagrange equation using a variational approach [40]. In this way, we verify that both the rotations around X and Z axes are robust against Ω-error since Equation (19) is satisfied. In Figure 2, we show the average fidelity of X and T gate against Ω-error, which also proves the robustness of geometric gates.

**Figure 3:**
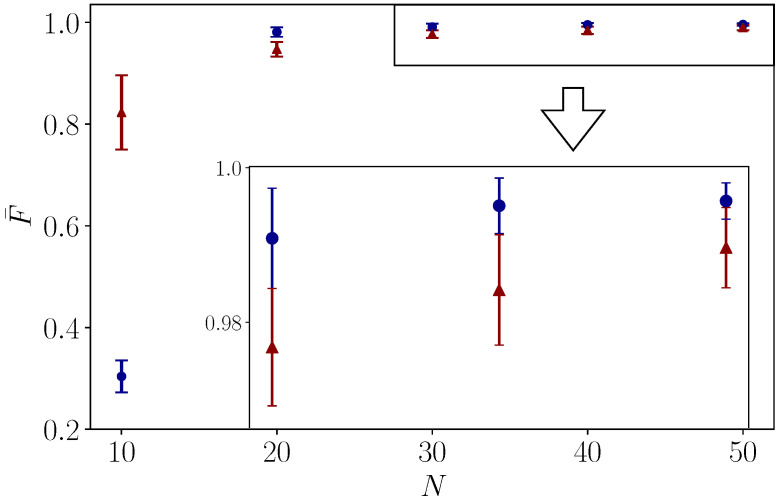
Digital quantum simulation of X (red line) and T gate (blue line) with different Trotter steps. A random systematic error $\delta_{\Omega }$ is generated by a Gaussian distribution of N(0,0.03), which verifies the robustness of nonadiabatic geometric quantum gates. We take 20 random configurations for each Trotter step, where the error bars denote the confidence intervals of 95%.

Concerning the digital quantum simulation, we assume that the Rx gate and Ry gate for simulating Ω(t)cosϕ(t) on $\sigma_{x}$ and −Ω(t)sinϕ(t) on $\sigma_{y}$ are perfect, which are physically realized by digitized external driving field. Accordingly, the performance of the digitized geometric gate depends on the number of Trotter steps. In Figure 3, we test the average fidelity of X and T gates digitized by different Trotter steps, being driven by external fields under randomized Ω-errors, as more evidence for verifying the robustness of STA inspired nonadiabatic geometric gates. In order to simulate the dynamics more precisely, one needs a larger gate number for reducing the Trotter error, treating Rx and Ry gates as the basic building blocks at the same time. For constructing the gates themselves, one has to modularize them by digital pulses (see Figure 1a,b) instead of directly implementing the circuits for simulation (see Appendix A), since Rx and Ry gates are not perfect in real devices. This suggests that the realistic architecture of a superconducting circuit for implementing the quantum gates with shortcut pulses will be very interesting for further investigation elsewhere. Another issue is that one will meet a scalability problem if a quantum algorithm is executed by geometric gates, which are substituted by circuit blocks that simulate the according dynamics.

## 4. Discussion

We have shown that one can construct nonadiabatic geometric gates via invariant-based STA. Here we analyze another approach, which employs CD terms to speed up and stabilize the evolution. A general two-level driving Hamiltonian is given by

(20) $$H_{d}\left(t\right)=h\left(t\right)\cdot \sigma ,$$

where σ denotes Pauli matrices, which the CD term that cancels diabatic transition reads

(21) $$H_{\mathrm{CD}}\left(t\right)=\frac{1}{{2|h\left(t\right)|}^{2}}\left[h\left(t\right)\times \dot{h}\left(t\right)\right]\cdot \sigma ,$$

yielding the trivial expression as,

(22) $$\begin{array}{c} H_{\mathrm{CD}}\left(t\right)=-\frac{1}{8}\dot{\phi }\left(t\right)\sigma_{z}, \end{array}$$

by using Equation (3) that gives $h\left(t\right)=\left(\Omega (t)\cos\phi (t),-\Omega (t)\sin\phi (t),0\right)$. The total Hamiltonian becomes

(23) $$H_{\mathrm{total}}\left(t\right)=\Omega \left(t\right)\cos\phi \left(t\right)\sigma_{x}-\Omega \left(t\right)\sin\phi \left(t\right)\sigma_{y}-\frac{1}{8}\dot{\phi }\left(t\right)\sigma_{z},$$

which introduces new boundary conditions $\dot{\phi }\left(0\right)=\dot{\phi }\left(T\right)=0$ since the CD term should not affect the system at the beginning and the end. In this way, one directly design Ω(t) and ϕ(t), evolving the total Hamiltonian with detuning for canceling the diabatic transition instead of designing θ(t) and β(t) based on invariant theory. However, we clarify that the application of a CD term for constructing nonadiabatic geometric gates is not as straightforward as invariant-based STA since nullifying dynamical phases by analytically designed control pulses is much harder. The digital quantum simulation of its dynamics in superconducting circuits is feasible, where similar shortcuts for digitized adiabatic quantum computing in single and multiple spin systems are experimentally implemented [47].

For the universal geometric quantum computation, we need a two-qubit geometric gate, which can also be accelerated by STA in a similar way. We assume that the two-qubit gate physically operates two coupled transmon qubits A and B with frequency $\omega_{A,B}$, frequency difference $\Delta =\omega_{A}-\omega_{B}$, and anharmonicity $\alpha_{A,B}$. The recent experiment [18] suggests that a time-dependent coupling g(t) can be realized by the longitudinal driving field with a fixed frequency ν, tunable phase ϕ(t), and amplitude λ(t). Hence, the effective Hamiltonian that describes the coupled transmon qubits is

(24) $$\begin{array}{c} H_{\mathrm{trans}}\left(t\right)=g{[|10\rangle }_{AB}\langle 01|e^{i\Delta t}+\sqrt{2}{|11\rangle }_{AB}\langle 02|e^{i(\Delta +\alpha_{B})t}+\sqrt{2}{|20\rangle }_{AB}\langle 11|e^{i(\Delta -\alpha_{A})t}]e^{-i\lambda (t)\sin[\nu t+\phi (t)]}. \end{array}$$

By letting $\nu =\Delta -\alpha_{A}$ under a weak coupling regime, a reduced two-level Hamiltonian in subspace ${\{|11\rangle }_{AB}$ and ${|20\rangle }_{AB}\}$ can be derived as

(25) $$H_{\mathrm{reduced}}\left(t\right)=\frac{1}{2}\left(\begin{array}{cc} 0 & 2\sqrt{2}gJ_{1}\left[\lambda \left(t\right)\right]e^{i\phi (t)}\\ 2\sqrt{2}gJ_{1}\left[\lambda \left(t\right)\right]e^{-i\phi (t)} & 0 \end{array}\right),$$

where $J_{1}\left[\lambda \left(t\right)\right]$ denotes the Bessel function of the first kind. The reduced Hamiltonian has the same form of Equation (3), which can also be inversely engineered for constructing two-qubit geometric gates. With computational bases be ${|00\rangle }_{AB}$, ${|01\rangle }_{AB}$, ${|10\rangle }_{AB}$, and ${|1\rangle }_{AB}$, one can accumulate a geometric phase $e^{i\gamma }$ on ${|11\rangle }_{AB}$ if the same protocol for rotation around the Z axis is applied, resulting in a control-PHASE gate as ${\mathrm{CU}}_{1}\left(\gamma \right)=\mathrm{diag}\left(1,1,1,e^{i\gamma }\right)$. In this way, one has the universal gate set for geometric quantum computing, realizing quantum error correction physically, which could be an alternative approach to fault-tolerant quantum computation.

## 5. Conclusions

In summary, we have developed the STA protocols for designing the fast and robust geometric quantum gates, by focusing on digital simulation in superconducting circuits for improving gate performance. Derived from the JC model with RWA, we obtain an effective two-level Hamiltonian describing a qubit driven by controllable pulses in the $\sigma_{x}$ and $\sigma_{y}$ direction. Invariant-based inverse engineering is employed to design the STA protocols, being simulated by quantum circuits, outputting high fidelity by canceling the transition induced from the systematic errors. Furthermore, we discuss the application of CD driving as another generally practiced STA, and extend the STA-inspired geometric gate to two qubits, which leads to a universal gate set as well.

## Appendix A. Digital Simulation by Suzuki-Trotter Expansion

Here we describe the details on the digitized process by using Suzuki–Trotter expansion, and its digital simulation in the quantum circuits. We use the asymptotic approximation theorem, i.e., Suzuki–Trotter formula [48]

(A1) $$\lim_{N\to \infty }{\left(e^{iAt/N}e^{iBt/N}\right)}^{N}=e^{i(A+B)t},$$

which is true even if *A* and *B* do not commute. Now we only consider the case of *A* and *B* being the Hermitian matrices. Modifications to Trotter’s formula provide a way to derive the higher-order approximations for performing quantum simulation

(A2) $$e^{i(A+B)\Delta t}=e^{iA\Delta t}e^{iB\Delta t}+O\left(\Delta t^{2}\right).$$

For the *N* trotter steps, we can decompose Equation (3) into *N* parts, which has the following form, $H=\sum_{k=1}^{N}H_{k}$, with

(A3) $$H_{k}=\frac{\hbar }{2}\left(\begin{array}{cc} 0 & \Omega_{k}\;e^{i\phi_{k}}\\ \Omega_{k}\;e^{-i\phi_{k}} & 0 \end{array}\right),$$

where $\Omega_{k}=\Omega \left(k\frac{t}{N}\right)$ and $\phi_{k}=\phi \left(k\frac{t}{N}\right)$. In this manner, the time evolution of the whole Hamiltonian, *H*, can be rewritten as

(A4) $$e^{-iHt}\approx \;\prod_{1}^{N}e^{-iH_{k}\frac{t}{N}}.$$

Then we finally decompose $H_{k}$ in the form of Pauli matrices, by setting $M=\Omega_{k}\;e^{i\phi_{k}}$. With the help of $H_{k}=Re\left(M\right)\sigma_{x}+Im\left(M\right)\sigma_{y}$, we can obtain

(A5) $$R_{x}\left(\theta \right)=\left(\begin{array}{cc} cos\frac{\theta }{2} & -isin\frac{\theta }{2}\\ -isin\frac{\theta }{2} & cos\frac{\theta }{2} \end{array}\right)\equiv e^{-i\frac{\theta }{2}\sigma_{x}},\;\;R_{y}\left(\theta \right)=\left(\begin{array}{cc} cos\frac{\theta }{2} & -sin\frac{\theta }{2}\\ sin\frac{\theta }{2} & cos\frac{\theta }{2} \end{array}\right)\equiv e^{-i\frac{\theta }{2}\sigma_{y}}.$$

In the main text, the time evolution of Hamiltonian *H* can be digitized by using such $R_{x}\left(\theta \right)$ and $R_{y}\left(\theta \right)$ gates. Accordingly, we implement the circuit on the platform of IBM Qiskit [49]. More details with error analysis are discussed, see recent work [47] as well.

## References

[1] Nielsen M.A., Chuang I.L. (2000). Quantum Computation and Quantum Information.

[2] Berry M.V. (1984). Quantal phase factors accompanying adiabatic changes. Proc. R. Soc. Lond. Ser. A.

[3] Wilczek F., Zee A. (1984). Appearance of gauge structure in simple dynamical systems. Phys. Rev. Lett..

[4] Aharonov Y., Anandan J. (1987). Phase change during a cyclic quantum evolution. Phys. Rev. Lett..

[5] Zanardi P., Rasetti M. (1999). Holonomic quantum computation. Phys. Lett. A.

[6] Sjöqvist E. (2008). Trend: A new phase in quantum computation. Physics.

[7] Zhu S.-L., Zanardi P. (2005). Geometric quantum gates that are robust against stochastic control errors. Phys. Rev. A.

[8] Leek P.J., Fink J.M., Blais A., Bianchetti R., Göppl M., Gambetta J.M., Schuster D.I., Frunzio L., Schoelkopf R.J., Wallraff A. (2007). Observation of Berry’s phase in a solid-state qubit. Science.

[9] Filipp S., Klepp J., Hasegawa Y., Plonka-Spehr C., Schmidt U., Geltenbort P., Rauch H. (2009). Experimental Demonstration of the Stability of Berry’s Phase for a Spin- Particle. Phys. Rev. Lett..

[10] Solinas P., Sassetti M., Truini T., Zanghí N. (2012). On the stability of quantum holonomic gates. New J. Phys..

[11] Kelly J., Barends R., Fowler A.G., Megrant A., Jeffrey E., White T.C., Sank D., Mutus J.Y., Campbell B., Chen Y. (2015). State preservation by repetitive error detection in a superconducting quantum circuit. Nature.

[12] Takita M., Cross A.W., Córcoles A.D., Chow J.M., Gambetta J.M. (2017). Experimental demonstration of fault-tolerant state preparation with superconducting qubits. Phys. Rev. Lett..

[13] Rosenblum S., Reinhold P., Mirrahimi M., Jiang L., Frunzio L., Schoelkopf R.J. (2018). Fault-tolerant detection of a quantum error. Science.

[14] Falci G., Fazio R., Palma G.M., Siewert J., Vedral V. (2000). Detection of geometric phases in superconducting nanocircuits. Nature.

[15] Leibfried D., DeMarco B., Meyer V., Lucas D., Barrett M., Britton J., Itano W.M., Jelenković B., Langer C., Rosenband T. (2003). Experimental demonstration of a robust, high-fidelity geometric two ion-qubit phase gate. Nature.

[16] Cui J.-M., Ai M.-Z., He R., Qian Z.-H., Qin X.-K., Huang Y.-F., Zhou Z.-W., Li C.-F., Tu T., Guo G.-C. (2019). Experimental demonstration of a robust, high-fidelity geometric two ion-qubit phase gate. Sci. Bull..

[17] Zhao P.-Z., Dong Z., Zhang Z., Guo G., Tong D.-M., Yin Y. (2019). Experimental realization of nonadiabatic geometric gates with a superconducting Xmon qubit. arXiv.

[18] Chu J., Li D., Yang X., Song S., Han Z., Yang Z., Dong Y., Zheng W., Wang Z., Yu X. (2020). Realization of Superadiabatic Two-Qubit Gates Using Parametric Modulation in Superconducting Circuits. Phys. Rev. Appl..

[19] Xu Y., Hua Z., Chen T., Pan X., Li X., Han J., Cai W., Ma Y., Wang H., Song Y.-P. (2020). Experimental implementation of universal nonadiabatic geometric quantum gates in a superconducting circuit. Phys. Rev. Lett..

[20] Wang X.-B., Keiji M. (2001). Nonadiabatic conditional geometric phase shift with NMR. Phys. Rev. Lett..

[21] Zhu S.-L., Wang Z.-D. (2002). Implementation of universal quantum gates based on nonadiabatic geometric phases. Phys. Rev. Lett..

[22] Chen T., Xue Z.-Y. (2018). Nonadiabatic geometric quantum computation with parametrically tunable coupling. Phys. Rev. Appl..

[23] Chen X.-Y., Li T., Yin Z.-Q. (2019). Nonadiabatic dynamics and geometric phase of an ultrafast rotating electron spin. Sci. Bull..

[24] Sjöqvist E., Tong D.M., Andersson L.M., Hessmo B., Johansson M., Singh K. (2012). Non-adiabatic holonomic quantum computation. New J. Phys..

[25] Xu G.-F., Zhang J., Tong D.M., Sjöqvist E., Kwek L.-C. (2012). Nonadiabatic holonomic quantum computation in decoherence-free subspaces. Phys. Rev. Lett..

[26] Zheng S.-B., Yang C.-P., Nori F. (2016). Comparison of the sensitivity to systematic errors between nonadiabatic non-Abelian geometric gates and their dynamical counterparts. Phys. Rev. A.

[27] Jing J., Lam C.H., Wu L.A. (2017). Non-Abelian holonomic transformation in the presence of classical noise. Phys. Rev. A.

[28] Santos A.C. (2018). Quantum gates by inverse engineering of a Hamiltonian. J. Phys. B At. Mol. Opt. Phys..

[29] Liu B.-J., Song X.-K., Xue Z.-Y., Wang X., Yung M.-H. (2019). Plug-and-Play Approach to Nonadiabatic Geometric Quantum Gates. Phys. Rev. Lett..

[30] Li S., Chen T., Xue Z.-Y. (2020). Fast holonomic quantum computation on superconducting circuits with optimal control. Adv. Quantum Technol..

[31] Xu J., Li S., Chen T., Xue Z.-Y. (2020). Nonadiabatic geometric quantum computation with optimal control on superconducting circuits. Front. Phys..

[32] Yan T., Liu B.-J., Xu K., Song C., Liu S., Zhang Z., Deng H., Yan Z., Rong H., Huang K. (2019). Experimental realization of nonadiabatic shortcut to non-Abelian geometric gates. Phys. Rev. Lett..

[33] Ai M.-Z., Li S., Hou Z., He R., Qian Z.-H., Xue Z.-Y., Cui J.-M., Huang Y.-F., Li C.-F., Guo G.-C. (2020). Experimental Realization of Nonadiabatic Holonomic Single-qubit Quantum Gates with Optimal Control in a Trapped Ion. arXiv.

[34] Torrontegui E., Ibánez S., Martínez-Garaot S., Modugno M., del Campo A., Guéry-Odelin D., Ruschhaupt A., Chen X., Muga J.G. (2013). Shortcuts to adiabaticity. Adv. Atom. Mol. Opt. Phys..

[35] Guéry-Odelin D., Ruschhaupt A., Kiely A., Torrontegui E., Martínez-Garaot S., Muga J.G. (2019). Shortcuts to adiabaticity: Concepts, methods, and applications. Rev. Mod. Phys..

[36] Berry M.V. (2009). Transitionless quantum driving. J. Phys. A Math. Theor..

[37] Chen X., Lizuain I., Ruschhaupt A., Guéry-Odelin D., Muga J.G. (2010). Shortcut to adiabatic passage in two-and three-level atoms. Phys. Rev. Lett..

[38] Chen X., Ruschhaupt A., Schmidt S., del Campo A., GuéryOdelin D., Muga J.G. (2010). Fast Optimal Frictionless Atom Cooling in Harmonic Traps: Shortcut to Adiabaticity. Phys. Rev. Lett..

[39] Lewis H.R., Riesenfeld W.B. (1969). An exact quantum theory of the time-dependent harmonic oscillator and of a charged particle in a time-dependent electromagnetic field. J. Math. Phys..

[40] Ruschhaupt A., Chen X., Alonso D., Muga J.G. (2012). Optimally robust shortcuts to population inversion in two-level quantum systems. New J. Phys..

[41] Lu X.-J., Chen X., Ruschhaupt A., Alonso D., Guérin S., Muga J.G. (2013). Fast and robust population transfer in two-level quantum systems with dephasing noise and/or systematic frequency errors. Phys. Rev. A.

[42] Munuera-Javaloy C., Ban Y., Chen X., Casanova J. (2020). Robust Detection of High-Frequency Signals at the Nanoscale. arXiv.

[43] Zahedinejad E., Ghosh J., Sanders B.C. (2016). Designing High-Fidelity Single-Shot Three-Qubit Gates: A Machine-Learning Approach. Phys. Rev. Appl..

[44] Ding Y., Ban Y., Martín-Guerrero J.D., Solano E., Casanova J., Chen X. (2020). Breaking Adiabatic Quantum Control with Deep Learning. arXiv.

[45] Chen X., Torrontegui E., Muga J.G. (2011). Lewis-Riesenfeld invariants and transitionless quantum driving. Phys. Rev. A.

[46] Daems D., Ruschhaupt A., Sugny D., Guérin S. (2013). Robust quantum control by a single-shot shaped pulse. Phys. Rev. Lett..

[47] Hegade N.N., Paul K., Ding Y., Sanz M., Albarrán-Arriagada F., Solano E., Chen X. (2020). Shortcuts to Adiabaticity in Digitized Adiabatic Quantum Computing. arXiv.

[48] Suzuki M. (1976). Generalized Trotter’s formula and systematic approximants of exponential operators and inner derivations with applications to many-body problems. Commun. Math. Phys..

[49] IBM Quantum Experience. https://www.research.ibm.com/ibm-q/.

